# Atypical Multiple Sclerosis Overlapping Features of Neuromyelitis Optica Spectrum Disorders (NMOSD)

**DOI:** 10.1002/ccr3.70608

**Published:** 2025-07-06

**Authors:** Sepideh Paybast, Ali Emami, Nasim Rezaeimanesh, Abdorreza Naser Moghadasi

**Affiliations:** ^1^ Multiple Sclerosis Research Center, Neuroscience Institute Tehran University of Medical Sciences Tehran Iran

**Keywords:** atypical presentation, multiple sclerosis, neuromyelitis optica spectrum disorders, overlap syndrome

## Abstract

We aim to discuss the importance of accurately diagnosing atypical inflammatory demyelinating diseases (IDD), particularly neuromyelitis optica spectrum disorders (NMOSD), which exhibit similar pathological characteristics to multiple sclerosis (MS). An accurate diagnosis is crucial as the disease‐modifying treatments (DMTs) used for MS can be ineffective or even exacerbate NMOSD. Our case was a 20‐year‐old man who presented with acute quadriparesis and hyperreflexia. Brain and cervical MRI revealed several T2‐weighted hyperintensities in the periventricular and corticomedullary junction areas. The CSF analysis showed six oligoclonal bands restricted to the CSF, and the serum AQP4‐IgG was negative. The patient was diagnosed with an atypical relapsing and remitting MS based on the 2017 revised McDonald criteria and was treated with intravenous methylprednisolone and therapeutic plasma exchange. Eventually, his EDSS score improved from 8.5 to 3 after treatment with rituximab. Accurate diagnosis of atypical cases of IDD is not only to prevent potential harm associated with misdirected therapies but also to promptly initiate the most effective treatment. Vigilance for diagnostic red flags in MS is particularly important given its clinical and radiographic overlap with NMOSD.

**Summary**

- The presented case highlights the importance of accurately diagnosing atypical inflammatory demyelinating diseases (IDD), such as NMOSD, which can present similarly to MS.
- According to the revised 2017 McDonald criteria for the diagnosis of MS, NMOSD should be considered in all patients with atypical features.
- This case emphasizes the significance of paying attention to red flags; the presence of these red flags underscores the need for further investigation and prudence when diagnosing MS.
- The red flags include a thunderclap headache preceded by myelitis, the severity of the myelitis leading to respiratory dysfunction, which is not typical in MS, and partial response to intravenous methylprednisolone (IVMP) and therapeutic plasma exchange (TPE).

## 1 Introduction

Most atypical inflammatory demyelinating diseases (IDD) exhibit pathological characteristics comparable to multiple sclerosis (MS). However, the presence of MS red flags makes it a clinical challenge with important treatment implications. In this regard, neuromyelitis optica spectrum disorders (NMOSD) are of great significance to distinguish from MS, especially during the initial presentation [1, 2]. Since most disease‐modifying drugs (DMDs) for MS can be ineffective or even exacerbate NMOSD, an accurate diagnosis of atypical cases remains crucial to prevent potential harm associated with misdirected therapies but also to promptly initiate the most effective treatment.

## 2 Case History

### 2.1 Clinical Presentation

A 20‐year‐old previously healthy man developed three episodes of generalized non‐thunderclap headache in a self‐remitting fashion a week before referral. The last episode was accompanied by acute quadriparesis, which progressed to respiratory discomfort. He reported no associated sensory complaints, band‐like sensations, or sphincter impairment. There was no relevant history of substance use, infection, or recent vaccination.

On examination, he was alert, conscious, and oriented. His vital signs revealed a body temperature of 36.5°C, blood pressure of 115/80 mmHg, respiratory rate of 26 breaths per min, pulse rate of 85 beats per min, and oxygen saturation of 96%. The meningeal irritation signs were negative. The neurological examination was notable for quadriplegia with the Medical Research Council muscle strength scale (MRC) grade 1. Generalized hyperreflexia, bilateral Hoffmann sign, and upgoing plantar reflexes were also evident. No sensory level was obtained.

## 3 Methods

### 3.1 Investigation and Treatment

**FIGURE 1:**
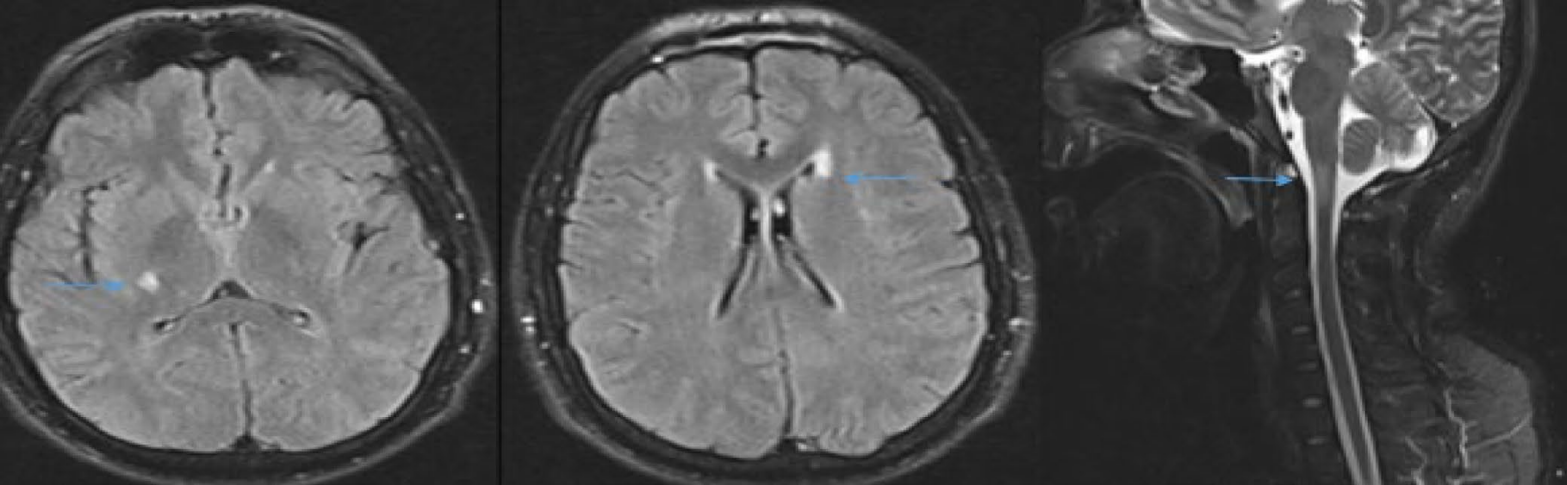
The MRI shows a FLAIR hyperintensity in the right internal capsule (left), sub ependymal periventricular area along the left lateral ventricle (middle), and a STIR cervicomedullary junction lesion in the sagittal image (right).

The initial investigations were normal, including brain computed tomography (CT) and routine biochemistry analysis. With a suspicion of acute myelopathy, the patient underwent brain and cervical spinal magnetic resonance imaging (MRI), which revealed a few ovoid T2 periventricular and right internal capsule hyper‐intense lesions and a short central T2 cervicomedullary junction hyperintense lesion (Figure 1). Cerebrospinal fluid (CSF) analysis demonstrated normal glucose levels, cell counts, and protein concentrations. Six oligoclonal bands (OCBs) were identified, exclusively restricted to the CSF. Comprehensive autoimmune antibody testing, including cell‐surface/synaptic antibodies (anti‐NMDA receptor [NMDAR], anti‐leucine‐rich glioma‐inactivated 1 [LGI1], anti‐contactin‐associated protein‐like 2 [CASPR2], anti‐α‐amino‐3‐hydroxy‐5‐methyl‐4‐isoxazolepropionic acid receptor [AMPA], anti‐γ‐aminobutyric acid receptor [GABA_B_ *R*], anti‐glutamic acid decarboxylase 65 [GAD65], anti‐dipeptidyl‐peptidase‐like protein 6 [DPPX], and anti‐IgLON5) and paraneoplastic antibodies (e.g., anti‐Hu, anti‐Yo), yielded negative results. Serologic evaluations were unremarkable, encompassing: aquaporin‐4 (AQP4) and myelin oligodendrocyte glycoprotein (MOG) antibodies (via fixed cell‐based assays); vasculitis/autoimmune panels: antinuclear antibody (ANA), anti‐double‐stranded DNA (dsDNA), lupus anticoagulant, anti‐β2‐glycoprotein‐I, anti‐cardiolipin, anti‐Smith (Sm), anti‐ribosomal P, perinuclear‐antineutrophil cytoplasmic antibody (p‐ANCA), cytoplasmic‐ANCA (c‐ANCA), and HLA‐B51, and infectious serologies: hepatitis B/C, HIV, tuberculosis (interferon‐gamma release assay), brucellosis, and enterovirus PCR. Notably, all diagnostic investigations were completed prior to the initiation of treatment.

In terms of treatment, he was admitted to the intensive care unit (ICU) and received concomitant intravenous methylprednisolone (IVMP) (7 g) and therapeutic plasma exchange (TPE) (7 sessions of 1.5 L).

## 4 Results

### 4.1 Outcome and Follow‐Up

The patient demonstrated significant clinical improvement, with his Expanded Disability Status Scale (EDSS) score decreasing from 8.5 to 5. Following evaluation using the 2017 McDonald criteria, the diagnosis was revised to atypical relapsing–remitting MS (RRMS). A high‐dose oral prednisolone regimen was initiated (50 mg daily for 1 week, tapered to 25 mg daily for 1 week, 12.5 mg daily for the third week, and 5 mg daily for the final week). For relapse prevention, rituximab (1 g total dose, divided into two 500 mg intravenous infusions administered 2 weeks apart) was introduced as a disease‐modifying therapy. After 3 months, the serum (AQP4) and (MOG) antibodies (via fixed cell‐based assays) were rechecked, which were still negative. At the 21‐week post‐treatment follow‐up, sustained clinical stability was observed, with a further reduction in EDSS to 3.

## 5 Discussion

According to the revised 2017 McDonald criteria for the diagnosis of MS, NMOSD should be considered in all patients with atypical features such as severe bilateral optic neuritis, severe brainstem involvement, longitudinally extensive transverse myelitis, and large cerebral lesions [1, 2].

Collectively, our case underscores the diagnostic challenges in distinguishing NMOSD from atypical MS, particularly in seronegative presentations. The presence of thunderclap headache preceding myelitis, respiratory dysfunction secondary to cervical cord involvement, and partial response to IVMP and TPE deviated from typical MS phenotypes. Thunderclap headache, though rare in MS, has been reported in NMOSD and other neuroinflammatory disorders [3]. Respiratory failure in MS is exceedingly uncommon and more suggestive of NMOSD‐associated LETM affecting the cervicomedullary junction [4]. Furthermore, the limited response to IVMP/TPE aligns with observations in NMOSD, where residual deficits often persist despite acute immunotherapy [1, 2]. In contrast, the presence of OCB and MRI findings was typical for MS. While current diagnostic frameworks for NMOSD and MOGAD prioritize antibody seropositivity, approximately 20% of NMOSD cases remain seronegative, complicating classification [1, 2]. Notably, OCB, a hallmark of MS, might rarely be positive in NMOSD, further blurring phenotypic boundaries [5]. Although serum cell‐based assays remain the gold standard for detecting AQP4‐IgG and MOG‐IgG, CSF testing for MOG‐IgG may serve as an adjunct in select cases with high clinical suspicion of MOGAD, albeit with caution due to lower specificity compared to serum [6]. These challenges emphasize the necessity of integrating clinical, radiographic, and longitudinal data to refine diagnosis and avoid therapeutic missteps. This diagnostic overlap has critical therapeutic consequences. Most MS DMDs are avoided in patients with NMOSD. In this regard, many experts favor immunosuppressive DMDs such as rituximab in overlapping syndromes, as we did in our patient [7, 8]. Further investigation is still required to discover the best management in overlapping syndromes.

Taking all considerations into account, our case highlights the importance of accurately diagnosing atypical IDD, such as seronegative NMOSD, which can present similarly to MS. An accurate diagnosis is essential to avoid inappropriate treatment with DMDs that can be ineffective or even exacerbate NMOSD. Given limited data on the overlapping features between double seronegative NMOSD and atypical MS, further investigations are required to discover the best management in such conditions.

## Author Contributions

**Sepideh Paybast:** methodology, writing – original draft, writing – review and editing. **Ali Emami:** methodology, writing – original draft, writing – review and editing. **Nasim Rezaeimanesh:** writing – review and editing. **Abdorreza Naser Moghadasi:** conceptualization, methodology, visualization, writing – original draft, writing – review and editing.

## Consent

The objectives of the study were explained to the patient and a written informed consent was obtained from the patient to publish this report in accordance with the journal's patient consent policy.

## Conflicts of Interest

The authors declare no conflicts of interest.

## Data Availability

The data is available upon a reasonable request to the corresponding author.
